# Impact of season on the association between muscle strength/volume and physical activity among community-dwelling elderly people living in snowy-cold regions

**DOI:** 10.1186/s40101-018-0186-6

**Published:** 2018-11-13

**Authors:** Junko Hasegawa, Hideki Suzuki, Taro Yamauchi

**Affiliations:** 1 School of Rehabilitation Sciences, Health Sciences University of Hokkaido, Ishikari-Tobetsu, Japan; 2 Graduate School of Rehabilitation Sciences, Health Sciences University of Hokkaido, Ishikari-Tobetsu, Japan; 3 Faculty of Health Sciences, Hokkaido University, Sapporo, Japan

**Keywords:** Physical activity, Daily step count, Community-dwelling elderly, Seasonal fluctuation

## Abstract

**Background:**

Although the benefits of physical activity are well-known, levels of physical inactivity are increasing in many countries. Physical activity, particularly for preventive care of the elderly, must be encouraged. The level of physical activity undertaken by people is influenced by season; however, little is known about seasonal fluctuations of physical activity and its relation to muscle strength/mass. Consequently, we clarified the association between physical activity levels and muscle strength/skeletal muscle mass during non-snowy and snowy seasons in northern Japan.

**Methods:**

Participants were community-dwelling elderly people aged 65 years or older living in Tobetsu, northern Japan. A 30-s chair-stand test (CS-30) and body composition measurements using bioelectrical impedance analysis were conducted prior to physical activity measurement using a three-dimensional acceleration sensor in both non-snowy and snowy seasons. Daily steps for the non-snowy and snowy seasons were compared using Welch’s *t* test. The association between the CS-30/skeletal muscle index and daily steps in both seasons was estimated by fitting multiple linear regression models, with age and sex as covariates.

**Results:**

Average daily step counts were significantly lower during the snowy season, compared to the non-snowy season (*P* < .01). The CS-30 in the snowy season alone was significantly associated with daily step counts. Multiple linear regression analyses results revealed that, for the same muscle strength in both seasons, the daily step counts during the snowy season were fewer than those during the non-snowy season.

**Conclusions:**

The muscle strength required to perform adequate physical activity depended on season. This study obtained basic knowledge to ensure health promotion for elderly people living in snowy-cold regions.

## Background

It is well-known that moderate physical activity promotes health. The benefits of physical activity have been extensively reported; physical activity improves quality of sleep, brain function, quality of life, and physical function and reduces depression and anxiety symptoms [1]. Research acknowledges the positive effect of physical activity on the risk reduction of cardiovascular disease and diabetes [2, 3]. However, physical inactivity levels are increasing in many countries [4]. In this era of super-aged societies, extension of healthy life expectancy is critical. Obviously, promoting the physical activity of elderly people is important, particularly in implementing preventive care strategies for elderly societies.

Outdoor physical activities are significantly influenced by season. Particularly, people who live in snowy regions find it difficult to engage in such activities in the snowy and cold winter environment. The influence of seasonal change on the activities of human beings has been studied, and people tend to be less active during winter in Iceland [5] and Japan [6]. People also tend to sleep longer in winter [7, 8].

Studies have extensively discussed the negative effects of inactivity; however, previous studies of aspects promoting physical activity mostly focused on behavior modification [9, 10] and provide little information on the relationship between physical capacity or function and physical activity. Cooper and colleagues [11] examined the associations between physical activity and hand grip strength among 60-year-old or older adults. They examined grip strength and moderate-to-vigorous physical activity (MVPA) twice in 4.5-year intervals and analyzed the mutual association between grip strength and MVPA at baseline and follow-up. It was found that grip strength at baseline and MVPA at follow-up were positively associated; on the contrary, MVPA at baseline and grip strength at follow-up were not associated. This indicates that a higher level of MVPA does not affect future muscle strength, whereas muscle strength is a determinant of future activity level. In general, physical activity decreases as age increases; however, among the elderly with sufficient muscle strength, the level of physical activity was maintained/barely decreased [11].

Regarding the association of body composition with physical activity, a high percentage of body fat was related to low physical activity [12, 13]; however, the relation between skeletal muscle mass and physical activity remains unclear. Another study reported that daily physical activity was not associated with handgrip strength and appendicular skeletal muscle mass [14]. A low skeletal muscle mass is included within the diagnostic criteria of sarcopenia. Hence, a better understanding of the association between skeletal muscle mass and physical activity should be obtained.

In general, people are likely to be inactive in winter; however, if their muscle strength ensures physical activity, the elderly with sufficient muscle strength can maintain their physical activity levels even in the snowy season. Indeed, the average daily step counts of participants in winter were larger than those in autumn [15]; however, the authors mentioned the possibility of this finding being influenced by the fact that participants were relatively healthy, and their instrumental activities of daily living scores were high. Little is known about the seasonal fluctuation of physical activity and its relation to muscle strength or muscle mass. It is important to clarify this point to devise effective strategies for the preventive care of community-dwelling elderly people in snowy-cold regions. This study clarifies the association between physical activity level and muscle strength/skeletal muscle mass during non-snowy and snowy seasons.

## Methods

Participants were community-dwelling elderly people aged 65 years or older who regularly attended senior club events in Tobetsu town, northern Japan. Tobetsu town is designated as a special heavy snowfall zone. The yearly average cumulative amount of snowfall is more than 8 m [16]. Prior to enrollment in this study, written informed consent was obtained from all participants.

### Measurement of lower limb muscle strength and volume

To estimate lower limb muscle strength, the 30-s chair-stand test (CS-30) was conducted as demonstrated in a previous study [17]. Skeletal muscle volume was measured with bioelectrical impedance analysis using InBody s10 (InBody Japan, Tokyo, Japan). Per the criterion provided by the Asian Working Group for Sarcopenia [18], the skeletal muscle mass index (SMI) was calculated. From March to July 2017, 59 participants underwent these measurements.

### Physical activity during the non-snowy season

To count participants’ daily steps from July to October 2017, the wearable fitness tracker Misfit Shine2 (Misfit, San Francisco, USA) was used. Participants wore the tracker for more than seven complete days. A dedicated application for the tracker was used to visualize daily step counts, and average daily steps were used for statistical analyses. In this measurement, 54 out of 59 people participated; however, the data of 4 people were lost due to bugs while synchronizing the data with the application.

### Physical activity during the snowy season

Participants attached not only the same wearable fitness tracker but also a Kenz Lifecorder GS (Suzuken, Nagoya, Japan) for insurance against data deficits during the snowy season. We explained the circumstances to participants and added the Lifecorder to monitor step counts. Participants were instructed to detach the Lifecorder only while bathing and sleeping. Although the data from the fitness tracker were used preferentially, there were nine instances of data deficit. To compensate, we checked the compatibility of step counts measured by the wearable fitness tracker and Lifecorder using Bland and Altman’s method [19]. Accordingly, 78 pairs of step count data were analyzed; the average and standard deviation of the differences in paired data (bias) were 265.8 and 1455.4, respectively. Although 95% of the data were within limits of agreement, a proportionality effect in which the fitness tracker was underestimated by over 3300 steps was observed. To convert data from the Lifecorder to those in the fitness tracker, a single regression analysis was conducted to obtain a regression equation and the step counts estimated by the regression equation were applied for the nine data deficits. Daily step counts for 29 people were obtained. Of those, 24 participated in both non-snowy and snowy season measurements. In other words, 26 people out of 50, who had their step counts measured in the non-snowy season, did not have their step counts measured in the snowy season.

### Statistical analyses

Age, body mass index (BMI), SMI, CS-30, and daily steps in the non-snowy and snowy seasons were compared using Welch’s *t* test. For both seasons, the associations between CS-30/SMI and daily steps were estimated by fitting multiple linear regression models, in which age and sex were set as covariates. All data were analyzed using JMP Pro 13.1.0 (SAS Inc., USA), with the significance level set as 5%.

## Results

Table 1 displays the characteristics of participants in both non-snowy and snowy seasons.

**Table 1:** Physical characteristics of participants in both non-snowy and snowy seasons

	Non-snowy season			Snowy season			*P*
	Total	Male	Female	Total	Male	Female	
Number of participants	50	13	37	29	8	21	–
Age	77.8 ± 5.3	79.5 ± 5.3	77.2 ± 5.3	78.4 ± 5.8	80.6 ± 5.5	77.5 ± 5.8	.65
BMI	24.5 ± 3.1	25.0 ± 2.8	24.3 ± 3.2	25.6 ± 3.5	26.8 ± 2.3	25.1 ± 3.8	.16
SMI	6.4 ± 1.0	7.4 ± 0.8	6.1 ± 0.8	6.6 ± 0.9	7.6 ± 0.6	6.2 ± 0.7	.43
CS-30	15.4 ± 4.3	13.3 ± 3.7	16.1 ± 4.3	15.6 ± 5.4	12.6 ± 4.5	16.8 ± 5.4	.86
Daily step counts	6.5 ± 3.2	4.6 ± 2.2	7.2 ± 3.2	4.2 ± 2.0	3.7 ± 2.3	4.4 ± 1.9	< .01

Daily step counts are displayed in units of 1000. *P* values are between both seasons among all participants

*BMI* body mass index, *SMI* skeletal muscle mass index, *CS-30* 30-s chair-stand test

No significant difference was observed between the two groups regarding mean age, BMI, SMI, and CS-30. The average daily step counts for Japanese people aged 70 years or older were 5744 steps and 4856 steps for men and women, respectively, according to a national survey held in 2016 [20]. Among men, daily step counts for both seasons were lower than the average. On the other hand, daily step counts for women during the non-snowy season constituted approximately 150% of the Japanese national average; however, the step counts drastically declined until they fell lower than the Japanese average during the snowy season. The average daily step counts were significantly lower during the snowy season, compared to the non-snowy season (*P* < .01). Table 2 displays the association between CS-30/SMI and daily step counts for both seasons.

**Table 2:** Effect of CS-30/SMI on daily step counts according to multiple linear regression analysis

	Daily steps (non-snowy season)				Daily steps (snowy season)			
	*β*	*P*	*β*	*P*	*β*	*P*	*β*	*P*
CS-30	0.20	.17			0.72	< .001		
SMI			0.22	.13			0.60	.03
Age	− 0.11	.42	− 0.22	.21	− 0.06	.95	− 0.36	.07
Sex	0.28	.05	0.45	.01	− 0.07	.63	0.50	.05
Adjusted *R*^2^	0.14		0.13		0.44		0.16	
Prob < *F*	0.02		0.02		0.00		0.07	

Note: Covariates included age, sex, and CS-30 or SMI. Two separate models were run for each season. Data are presented as standardized *β* (*β*) and *P* values (*P*). A negative *β* value indicates an inverse relationship

*CS-30* 30-s chair-stand test, *SMI* skeletal muscle mass index

On setting daily steps during the non-snowy season as a dependent variable, neither CS-30 nor SMI were significant predictors of daily step counts, although both the regression equations were significant. When the dependent variable was set as daily steps during the snowy season, the regression equation for SMI was non-significant (*P* = .07), although the standardized partial regression coefficients were relatively high (*β* = 0.60) and *P* = .03. For CS-30, the regression equation was significant, and CS-30 was the main component of the equation, as well. To simplify the association between muscle strength and daily step counts, Tables 3 and 4 display the correlation between CS-30/SMI and daily step counts, and the regression equation stratified by sex, since we judged that age had no influence according to the results demonstrated in Table 2.

**Table 3:** Effect of CS-30 on daily step counts according to single regression analysis stratified by sex

	Non-snowy		Snowy	
	Male	Female	Male	Female
Number of participants	13	37	8	21
*R*	0.08	0.27	0.87	0.64
*P*	.80	.10	.01	< .001
Intercepts	3966	3872	− 940	629
Gradients	47	205	363	224

**Table 4:** Effect of SMI on daily step counts according to single regression analysis stratified by sex

	Non-snowy		Snowy	
	Male	Female	Male	Female
Number of participants	13	37	8	21
*R*	0.19	0.12	0.15	0.38
*P*	0.54	0.4	0.72	0.08
Intercepts	543	4253	232	− 1965
Gradients	549	482	447	1021

As indicated by the multiple linear regression model, no correlation was observed during the non-snowy season, whereas a significant positive correlation was observed during the snowy season between physical activity and muscle strength. The single regression analysis indicated the same tendency as the multiple linear regression model. When daily step counts were the dependent variable, the intercepts were approximately 4000 for both male and female participants in the non-snowy season, whereas in the snowy season, they were approximately 600 for female participants and 1000 for male participants. If the possible values of CS-30 range 0–40, according to the judgment criteria, even when the regression equation of the non-snowy season was not considered significant, daily step counts in the snowy season were overwhelmingly lower than those during the non-snowy season for the same level of muscle strength.

## Discussion

Our results reveal that daily step counts during the snowy season were significantly lower than those during the non-snowy season. This supports the findings of most previous studies [21–24]. In this study, we prospectively examined the association between lower limb muscle strength/volume and daily steps. As a result, CS-30 and average daily step counts demonstrate a significant positive correlation only during the snowy season. Interestingly, this correlation was not observed during the non-snowy season. Although the SMI demonstrated a similar tendency as muscle strength, no significant difference was confirmed.

In the snowy season, the condition of roads completely differs from that in the non-snowy season; during the snowy season, roads are frozen or have a snowy surface. A person’s strategy of walking on such roads also differs from his or her normal gait. Regarding walking on frozen road surfaces, detailed kinematics were analyzed. The flexion/extension moment of rotation of the hip joint increases while walking on a slippery floor [25]. To avoid slipping on a frozen road surface, increased flexion/extension movement enables the floor reaction force to be adjusted vertically. This means that greater muscle strength of hip flexion/extension is required to avoid slipping on a frozen road. In addition, in a snowy season environment, people must push the snow aside using their legs. Moreover, rubber boots—which are often worn during the snowy season—and winter clothes tend to be heavier than normal clothing. By comprehensively considering these factors, we speculate that greater muscle strength is required for people to walk outside in snowy season.

Physical activity is essential for the promotion of health. Studies have reported various effects of physical activity, such as the reduction of mortality rate and risk of cardiovascular disease, metabolic diseases, obesity, falls, dementia, osteoporosis, and musculoskeletal diseases. The quantity–response association between physical activity and health was reported; however, even very-low-intensity physical activity is effective in promoting health. Engagement in physical activity is affected by factors such as pedestrian infrastructure and road safety [26]. In addition, studies have examined various types of interventions to promote physical activity [9, 10]; however, the association between physical capacity and activities of daily living has not been well investigated. This research gap was successfully addressed by the current study. As mentioned earlier, Cooper and colleagues [11] reported that older adults who maintained/improved their muscle strength were more likely to increase their levels of physical activity during follow-up, and those who increased their level of physical activity did not increase their muscle strength. The results of the current study reveal that muscle strength is a key factor that affects the level of physical activity of individuals, which coincides with the results reported by Cooper and colleagues. Low muscle strength is likely one of the causes of inactivity. Conversely, if we improve muscle strength with appropriate interventions, it may promote physical activity.

We showed that muscle weakness is a plausible cause of inactivity during winter; however, inactivity can occur for various reasons. The weather, such as temperature, rain, or wind, influences physical activity [27]. Further, regardless of season, people with a fear of falling tend to curtail activities [28]. To promote physical activity, the usage of a cane, non-slip shoes, or such items should be considered to offset individuals’ fear of falling. In addition, to understand how people seek necessary information to avoid falling while walking outdoors, especially on slippery road surfaces, it is vital to dispel people’s fear of falling. Suzuki and colleagues reported that elderly people tend to pay much more attention to other things rather than the walking path or their footing on an icy road [29]. Auditory information or sensations received from the sole of the foot are potential information to avoid falling as well as visual information. Such understanding of walking characteristics may enlighten elderly adults and prevent falling.

**Fig. 1:**
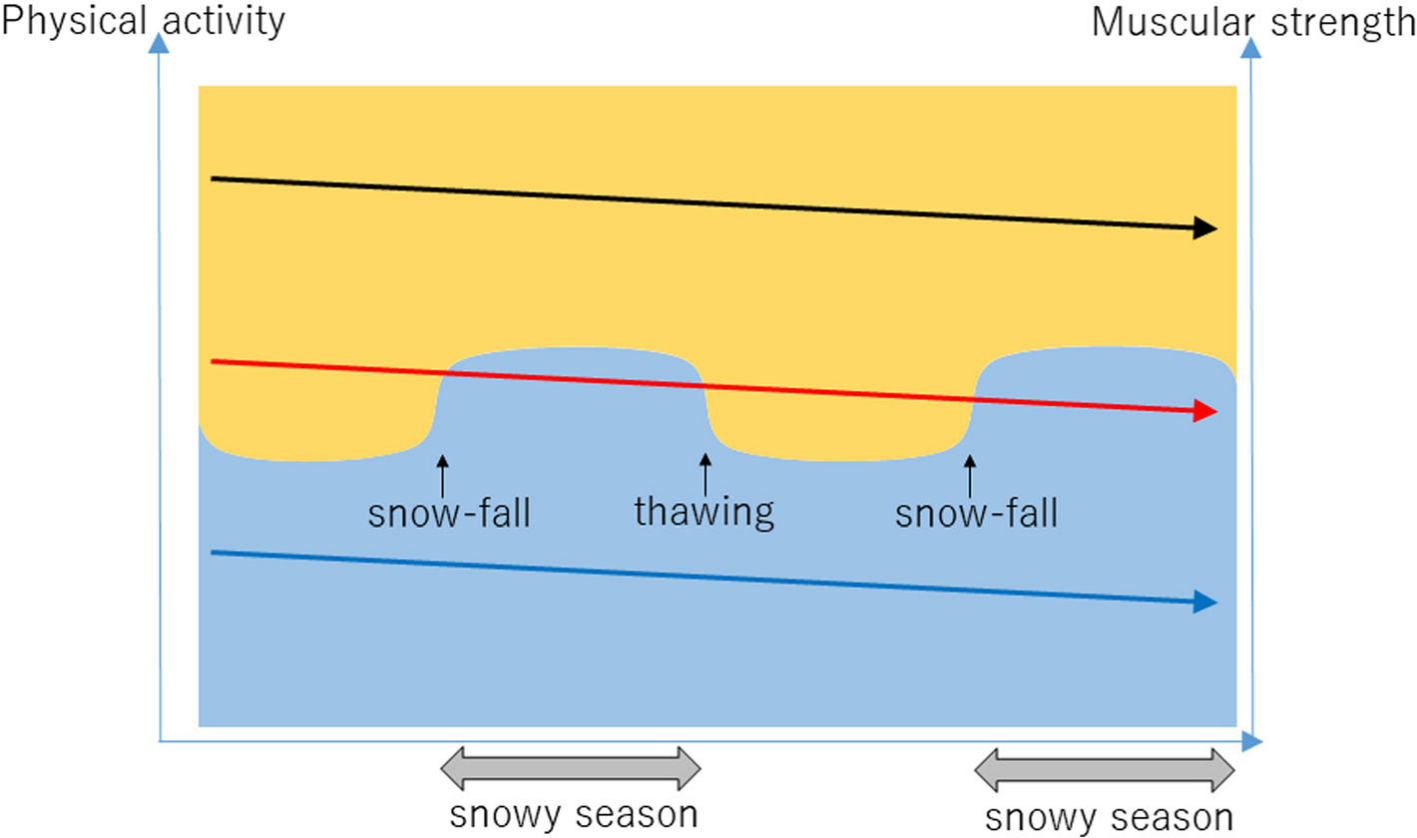
Hypothesis of seasonal fluctuation on muscle strength required to perform physical activity. The vertical axes represent muscle strength and physical activity. The background color refers to physical activity; the blue zone implies physically inactive, whereas the orange zone implies active. Three different levels of muscle strength are displayed using the three arrows in Fig. 1. The arrows represent individuals with different muscle strength levels; the black, blue, and red arrows represent adequate muscle strength, remarkably low muscle strength, and intermediate muscle strengths, respectively. The gentle downward slope of each arrow represents muscle weakness due to aging. Although these arrows may slope downward due to a certain event or the degree of slope may differ between individuals, the arrows are presented as even lines here. The black arrow indicates individuals who are physically active throughout the year, and, likewise, blue indicates those who are inactive. The individuals represented by the red arrow show seasonal fluctuations in physical activity, with adequate physical activity during the non-snowy season and inactivity during the snowy season

Since there was a seasonal difference in the association between lower limb muscle strength and daily step counts, we assume that the muscle strength required to perform sufficient physical activity depends on the season. In other words, adequate muscle strength guarantees sufficient physical activity regardless of the season; however, when muscle strength declines to a certain level, sufficient physical activity can be guaranteed in the non-snowy season alone. Schematically, Fig. 1 presents this hypothesis. The level of lower limb muscle strength required to be physically active is higher in the snowy, rather than the non-snowy, season, as mentioned above. On the premise of lower limb muscle strength weakening due to aging, when healthy elderly people reach a certain level of muscle strength, they can be physically active during the non-snowy season but be inactive during the snowy season. Although a healthy elderly person’s strength gradually weakens with the passage of time, activity in the non-snowy season, coupled with inactivity in the snowy season, illustrates the early stages of elderly frailty. In other words, seasonal fluctuations in physical activity among community-dwelling elderly people in snowy-cold regions could be a key factor in the early diagnosis of frailty. Moreover, this presents further implications for clarifying the methods/timing of intervention or resilience during the snowy and non-snowy seasons, although the hypothesis requires further study.

This study had some limitations. First, we had a small sample size; however, the study applies appropriate statistical analyses. Second, the use of two different instruments to measure physical activity levels required the use of suitable conversion methods. Third, physical activity was evaluated using daily steps alone, which does not reflect activity intensity. Fourth, alertness for falling might affect physical activity levels; however, this point was not considered in the current study. Based on these points, we intend to continue examining the influence of seasonal changes on physical activity among the elderly.

## Conclusions

In this study, we focused on the association between seasonal changes in physical activity and lower limb muscle strength/volume among the elderly. Similar to previous studies, we confirmed that the level of physical activity decreased during the snowy season. We hypothesized that there is a seasonal difference in the lower limb muscle strength that is required to maintain the level of physical activity. Further, we obtained basic knowledge to promote the health of elderly people living in snowy-cold regions.

## Abbreviations

BMI: Body mass index
CS-30: 30-s chair-stand test
MVPA: Moderate-to-vigorous physical activity
SMI: Skeletal muscle mass index
